# Gingival Stem Cell‐Conditioned Media and Low‐Level Laser Therapy Enhance Periodontal Ligament Stem Cells Function by Upregulating Wnt and TGF‐β Pathway Components: An In Vitro Study

**DOI:** 10.1002/cre2.70151

**Published:** 2025-05-26

**Authors:** Mohammed Y. Aljabri, Yaser A. Alhazmi, Salah A. Elsayyad, Shehabeldin M. Saber, Mohamed Shamel

**Affiliations:** ^1^ Department of Oral and Maxillofacial Surgery and Diagnostic Sciences, College of Dentistry Jazan University Jazan Saudi Arabia; ^2^ Dental Science Research Group, Health Research Centre of Excellence The British University in Egypt (BUE) El Sherouk City Egypt; ^3^ Department of Endodontics, Faculty of Dentistry The British University in Egypt (BUE) El Sherouk City Egypt; ^4^ Department of Endodontics, Faculty of Dentistry Ain Shams University Cairo Egypt; ^5^ Oral Biology Department, Faculty of Dentistry The British University in Egypt Cairo Egypt

**Keywords:** gingival stem cell‐conditioned media, low‐level laser therapy, osteogenic differentiation, PDLSCs, periodontal regeneration, TGF‐β signaling pathway, Wnt signaling pathway

## Abstract

**Objectives:**

This study investigated the synergistic effects of gingival stem cell conditioned media (GSC‐CM) and low‐level laser therapy (LLLT) on stimulating PDLSCs, explicitly focusing on the molecular basis of enhancing the Wnt and TGF‐β signaling pathways.

**Material and Methods:**

PDLSCs were isolated and GSC‐CM were prepared and characterized. Five groups were utilized: control, osteogenic induction medium (positive control), GSC‐CM, LLLT (980 nm, 1.5 J/cm²), and a combination of GSC‐CM with LLLT. Cell viability was assessed using the MTT assay on Days 1, 2, and 3. Osteogenic differentiation of PDLSCs was assessed using mineralization assays employing Alizarin Red staining, and molecular analyses of osteogenic markers (*RUNX2, ALP, OCN*) and signaling‐related genes (*CTNNβ1, TGFβ1*) conducted via RT‒PCR.

**Results:**

The findings revealed that the dual application of GSC‐CM and LLLT significantly enhanced cell viability and osteogenic differentiation of PDLSCc compared to the effects of the individual treatment modalities. Moreover, the study revealed elevated expression levels of osteogenic markers across all the experimental groups, specially the combination group, which showed the highest levels. Significantly, the combination treatment group displayed superior outcomes in this regard. The results also suggest potential activation of Wnt/β‐catenin and TGF‐β signaling components.

**Conclusions:**

This study highlights the significant effects of using GSC‐CM and LLLT to improve PDLSC survival and osteogenic differentiation.

**Abbreviations**
ALP: alkaline phosphatase
ATP: adenosine triphosphate
CTNNβ1: beta‐catenin
DMSO: dimethyl sulfoxide
GMSCs: gingival mesenchymal stem cells
G‐SCCM: gingival stem cell‐conditioned media
IL‐10: interleukin‐10
IL‐1β: interleukin 1 Beta
LLLT: low‐level laser therapy
MSC: mesenchymal stem cells
OCN: osteocalcin
PDLSCs: periodontal ligament stem cells
PRP: platelet‐rich plasma
ROS: reactive oxygen species
RT‐qPCR: real‐time quantitative polymerase chain reaction
RUNX2: runt‐related transcription factor 2
TGF‐β: transforming growth factor‐beta
TNF‐α: tumor necrosis factor‐alpha

## 1 Background

Periodontitis is a complex and chronic inflammatory oral disease that is widespread worldwide. Pathogenesis of periodontitis involves an interaction between oral bacteria, the immune system, and the tissues of the oral cavity (Sedghi et al. 2021). Periodontitis is associated with the destruction of periodontal tissues, including the gingiva, periodontal ligament (PDL), cementum, and alveolar bone. If periodontitis is not effectively controlled, it will gradually result in the loosening and eventual loss of teeth. This disease can have a substantial influence on dental health, requiring immediate intervention to prevent related problems (Tonetti et al. 2018; Kim and Amar 2006).

Traditionally, periodontitis is treated in clinical practice using mechanical debridement procedures, and maybe sometimes combined with the use of antibiotics and other medications (Manresa et al. 2018; Sanz et al. 2020; Hammami and Nasri 2021). Various alternatives have emerged in the field, including guided tissue regeneration, the use of platelet‐rich plasma (PRP), and the application of both natural and synthetic biomaterials, all aimed at regenerating periodontal tissues that have been lost (Villar and Cochran 2010; Sheikh et al. 2017). Nonetheless, many of these methods exhibit outcomes that are often inconsistent, unpredictable, and subject to considerable variation (Chen et al. 2010).

Stem cell‐mediated periodontal regeneration is a subject of great focus and interest (Sun et al. 2023). Various types of mesenchymal stem cells (MSCs) have been used to regenerate periodontal tissues (Di Vito et al. 2023). Nevertheless, it is crucial to acknowledge that stem cell‐based therapies face significant challenges, such as their source, yield, potential dedifferentiation during amplification, diminished regeneration efficacy, and the complexities associated with quality control when scaling up cell production. These obstacles represent important considerations in the ongoing development and application of stem cell‐based approaches for periodontal regeneration (Lin et al. 2021).

A cell‐free alternative method that is utilized in regenerative approaches is the use of stem cell‐conditioned media (SC‐CM). SC‐CM involves the extraction of proteins, growth factors, cytokines, and enzymes from cultured stem cells without the need for the cells themselves. These elements are utilized for therapy (Chen et al. 2019; El Moshy et al. 2020). Compared with cell‐based therapies, the use of SC‐CM offers numerous notable benefits. This approach decreases the likelihood of host immunological reactions by delivering necessary proteins instead of whole cells, thereby providing safer operation. SC‐CM can be stored for extended durations without the need for toxic cryopreservatives, ensuring improved stability and reduced risk. Furthermore, SS‐CM provides a cost‐effective option and simplifies the assessment of safety and efficacy, hence enhancing the efficiency of evaluating its therapeutic potential. This makes it an extremely promising approach in the field of regenerative medicine and numerous other medicinal applications (Kovach et al. 2015). Cumulative studies have revealed the various biological activities of SC‐CM, including angiogenesis (Burlacu et al. 2013), osteogenesis (Katagiri et al. 2015), chemotaxis (Inukai et al. 2013), immunomodulation (Yu et al. 2018), and cell growth and differentiation, which can facilitate periodontal regeneration (Lin et al. 2021).

Among the various types of MSCs derived from oral tissues, gingival mesenchymal stem cells (GMSCs) have demonstrated remarkable potential for clinical applications in bone reconstruction and repair (Shamel et al. 2024; Jin et al. 2015; Alhazmi et al. 2023; Tomar et al. 2010; Goriuc et al. 2023). Notably, both periodontal ligament stem cell‐conditioned medium (PDLSC‐CM) and GMSC‐conditioned medium (GMSC‐CM) have significant effects on periodontal regeneration (Fawzy El‐Sayed and Dörfer 2016). This is accomplished by decreasing the levels of inflammatory factors such as TNF‐α and IL‐1β while encouraging the upregulation of bone‐related indicators such as BSP‐II and Runx2. Furthermore, the GMSC‐CM group exhibited a notable increase in the expression of the anti‐inflammatory cytokine IL‐10 compared to the PDLSC‐CM group within a rat model of periodontal defects (Qiu et al. 2020). These findings underscore the promising role of GMSCs and their conditioned medium in advancing periodontal regeneration therapies.

Recently, laser‐assisted periodontal therapy has emerged as an innovative and minimally invasive method, garnering increasing attention. It has numerous benefits compared to conventional surgical approaches, such as decreased discomfort, expedited recovery, and reduced postoperative swelling (Everett et al. 2017; El Mobadder et al. 2023). Using specific laser wavelengths, scientists and healthcare professionals can stimulate the regenerative potential of MSCs, which play a vital role in the restoration of periodontal tissues affected by disease or trauma (Wu et al. 2023; Wang et al. 2022; Gholami et al. 2021).

Low‐level laser therapy (LLLT), also known as photobiomodulation therapy, employs low‐intensity lasers to activate cellular mechanisms within periodontal tissues (Alhazmi et al. 2023). These processes include encouraging cell growth, synthesizing collagen, and stimulating angiogenesis, all of which contribute to tissue mending and rejuvenation (Berni et al. 2023). Additionally, LLLT can facilitate the delivery of growth factors or biomaterials designed to amplify tissue regeneration. By establishing microchannels in tissues, lasers facilitate the permeation and absorption of these therapeutic substances, thereby fostering healing and regeneration (Pansani et al. 2017).

Although gingival stem cell conditioned media (GSC‐CM) and LLLT have shown promise for enhancing cellular functions, the synergistic effects of combining these treatments on PDLSCs have yet to be explored. This study represents a promising step toward more effective treatments for periodontal diseases and enhanced oral health. The null hypothesis in this context is that there is no difference or effect when using LLLT, GSC‐CM, or a combination of both on cell viability and the osteogenic differentiation potential of PDLSCs in vitro.

## 2 Methods

The following five experimental groups were used throughout the study:

1. **Control Group:** PDLSCs cultured in standard medium without any treatment.
2. **Osteogenic Group (Positive Control):** PDLSCs cultured in osteogenic differentiation medium.
3. **GSC‐CM Group:** PDLSCs treated with 100‐fold concentrated GSC‐CM.
4. **LLLT Group:** PDLSCs exposed to 980 nm low‐level laser therapy (LLLT) at 1.5 J/cm² for 60 s.
5. **GSC‐CM** + **LLLT Group:** PDLSCs first pretreated with GSC‐CM, then exposed to LLLT under the same conditions.

After providing informed consent, samples of healthy gingival tissue and periodontal tissues were collected from five patients aged 18–25 years following mandibular third molar extraction.

### 2.1 Isolation and Culture of GMSCs and PDLSCs

To isolate GMSCs, healthy gingival tissues were incised from around the extracted teeth. The tissues were then gently washed with phosphate‐buffered saline (PBS) and cut into ~1 mm³ fragments for enzymatic digestion.

For PDLSCs isolation, the central third of the extracted root portions were carefully scraped using a sterile blade before being placed into separate sterile Petri dishes. After being collected, these tissues were thinly sliced into pieces ~1 mm^3^ in size for enzymatic digestion.

Enzymatic digestion was used to extract GMSCs and PDLSCs in accordance with earlier research (Alhazmi et al. 2023; Saber et al. 2023). Briefly, the tissue pieces were submerged in a recently prepared collagenase type I and dispase II solution (Gibco, Thermo Fischer Scientific, USA). After 3 min of centrifugation at 200 × *g*, the cells were separated and reconstituted in complete culture media.

### 2.2 GMSC and PDLSC Characterization

The presence of MSC‐associated surface antigens was identified by flow cytometry. GMSCs and PDLSCs were incubated with primary antibodies against CD73, CD90, CD105, CD34, CD45, and HLA‐DR and then analyzed by a flow cytometer (Bakr et al. 2024).

### 2.3 Preparation and Concentration of GSC‐CM

GSC‐CM was made in accordance with earlier research (Qiu et al. 2020). In brief, GMSCs were grown in medium supplemented with 10% fetal bovine serum until they reached approximately 80% confluence. After that, serum‐free α‐MEM was added to the medium, and the cells were cultured for 48 h. To obtain GSC‐CM, the supernatants were filtered through 0.22‐μm filters. The collected GSC‐CM were subsequently concentrated 100‐fold using ultrafiltration centrifuge tubes (Ultra‐15 10 kD centrifugal filter, EMD Millipore, Billerica, MA, USA) at 5000 g and 4°C for 40 min, following the manufacturer's protocol. Control CM was prepared by incubating serum‐free α‐MEM for 48 h under the same conditions and concentrating it similarly. The PDLSCs were exposed to the concentrated GSC‐CM for 24 h before any additional treatments or experiments were applied, allowing sufficient time for the conditioned medium to exert its effects on the cells.

### 2.4 LLLT Protocol

PDLSCs, cultured in serum‐free α‐MEM, were irradiated using a GaALAs Diode laser system (K2 mobile laser, Hulaser, Seoul, Republic of Korea) that emitted light at a wavelength of 980 nm continuously, applying an energy density of 1.5 J/cm² over 60 s (Alhazmi et al. 2023).

### 2.5 MTT Assay

The MTT test was used to evaluate the viability of the PDLCS (Rady et al. 2024). In summary, 200 µL of media was added to each well of 96‐well tissue culture plates, and PDLSCs were seeded at a density of 3.5 × 104 cells/mL. Following a 24‐h incubation period, PDLSCs were subjected to LLLT, GSC‐CM, or a combination of the two treatments. The cells were then cultured for 1, 3, or 7 days. PDLSCs were incubated in MTT solution at 37°C for 3 h. The optical density was measured using a microplate reader, and the findings were calculated using a formula to determine the % viability (Rady et al. 2024; Sayed et al. 2023).

### 2.6 Osteogenic Differentiation

For osteogenic differentiation, PDLSCs were seeded in culture media until they reached 70% confluence. Afterward, the media was replaced with osteogenic media (Millipore Sigma). PDLSCs cultured in standard media served as a negative control, while PDLSCs cultured in osteogenic media only served as a positive control (Rady et al. 2024). The three experimental groups were subjected to LLLT, GSC‐CM, or their combination.

### 2.7 Mineralization Assay

Two weeks after the start of osteogenic induction, mineral deposition was evaluated using Alizarin Red (AR) staining. The stained mineralized nodules were examined after staining PDLSCs for 5 min with a 2% AR solution (Sigma‒Aldrich Corporation). An inverted microscope was used to obtain images. AR absorbance rates were determined using a microplate reader set at 405 nm to measure the degree of mineralization (Bakr et al. 2024).

### 2.8 Expression of Osteogenic Markers and Signaling Pathway Genes

The mRNA expression levels of osteogenic markers; Runt‐related transcription factor 2 (RUNX2), alkaline phosphatase (ALP) and osteocalcin (OC), the Wnt signaling pathway gene beta‐catenin (*CTNNβ1*) and the transforming growth factor‐*β*1 signaling pathway gene (*TGF‐β1*) were determined by real‐time quantitative polymerase chain reaction (RT‒qPCR). Gene expression was normalized against that of β‐actin (Rady et al. 2024). The primer sequences are listed in Table 1.

**Table 1:** Sequences of Primers used for RT‒PCR.

Gene	Forward Primer	Reverse Primer	Product size
*RUNX2*	GTTATGAAAAACCAAGTAGCCAGGT	GTAATCTGACTCTGTCCTTGTGGAT	157
*ALP*	GACCTCCTCGGAAGACACTC	TGAAGGGCTTCTTGTCTGTG	142
*OC*	CGCCTGGGTCTCTTCACTAC	CTCACACTCCTCGCCCTATT	120
*CTNNβ 1*	ATGGCTTGGAATGAGACTGCT	GGGTCCATACCCAAGGCATC	168
*TGF‐β1*	GGATACCAACTATTGCTTCAGCT	AGGCTCCAAATGTAGGGGCAGGG	189
*β‐actin*	TCCGTCGCCGGTCCACACCC	TCACCAACTGGGACGATATG	150

### 2.9 Statistical Analysis

The data obtained are presented as the mean values with corresponding standard deviations. Statistical assessments were conducted through two‐way analysis of variance (ANOVA) followed by post hoc Tukey tests utilizing GraphPad Prism v8.1.0 software (GraphPad Software, San Diego, CA). A significance level of *p* < 0.05 was considered to indicate statistical significance. All experiments were replicated five times and independently performed on at least three occasions.

## 3 Results

### 3.1 Isolation and Identification of PDLSCs and GMSCs

**Figure 1:**
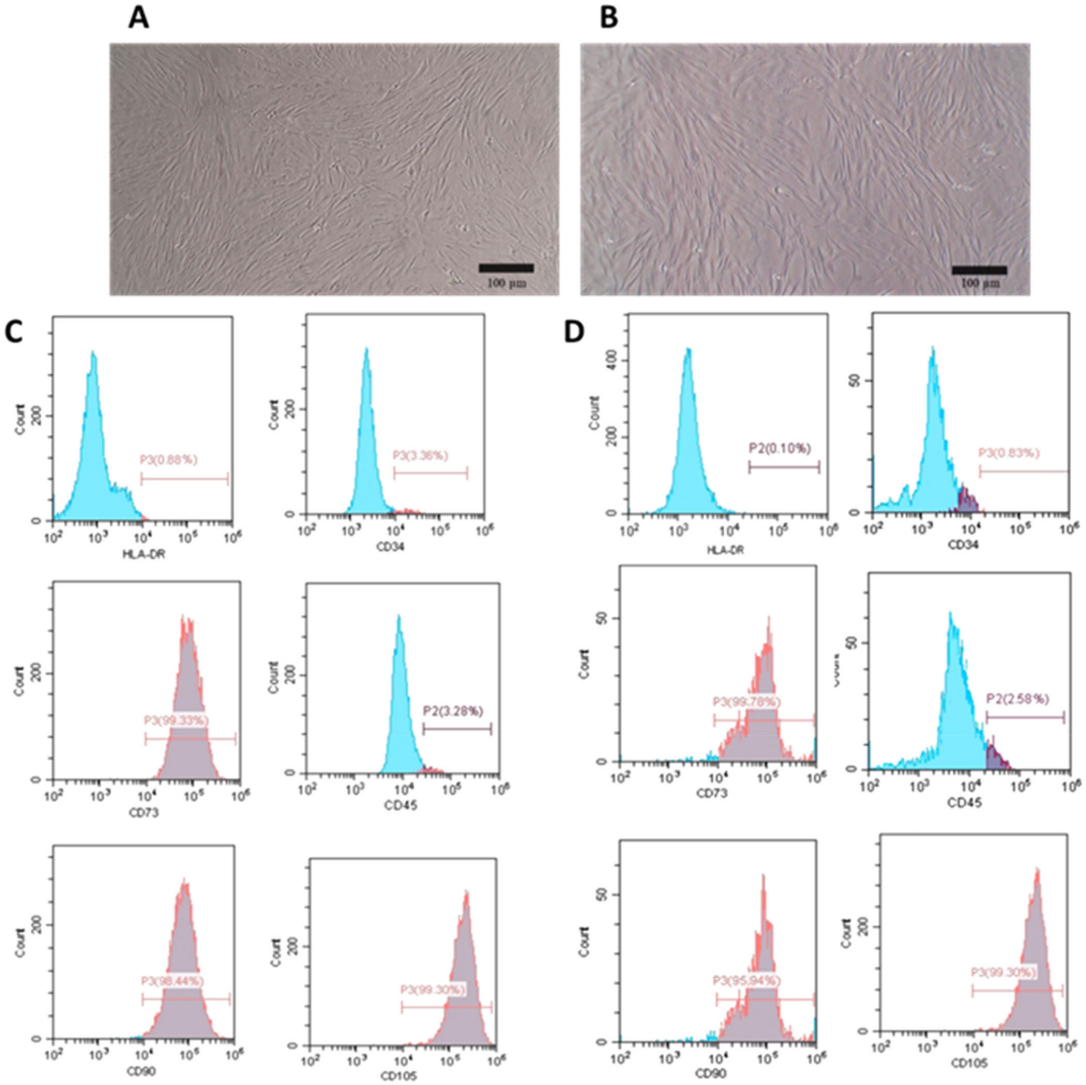
Characterization of MSCs. (A) Morphological appearance of the isolated PDLSCs. (B) Morphological appearance of the isolated GMSCs. (C) Flow cytometry analysis of PDLSCs. (D) Flow cytometry analysis of GMSCs.

PDLSCs and GMSCs exhibited an adherent, fibroblast‐like appearance characterized by elongated and spindle‐shaped morphology, as shown in Figure 1A,B. Flow cytometry analysis confirmed the presence of typical MSC‐associated surface markers, including CD73, CD90, and CD105. In contrast, the hematopoietic cell surface markers CD34 and CD45 were not detected (Figure 1C,D).

### 3.2 MTT Assay

On Day 1, all treatments started at the same baseline, which was normalized to 100% viability. By Day 3, all treatment groups exhibited an increase in cell viability compared to day 1. Among them, LLLT alone showed the least increase. Both the GSC‐CM and (GSC‐CM + LLLT) groups demonstrated greater viability, with the combination group (GSC‐CM + LLLT) showing a notably higher increase than GSC‐CM alone. By Day 7, cell viability increased further under all conditions. The combined treatment (GSC‐CM + LLLT) had the greatest effect on cell viability, while LLLT alone had the least effect on cell viability among the three treatments. However, viability still increased beginning on Day 3.

**Figure 2:**
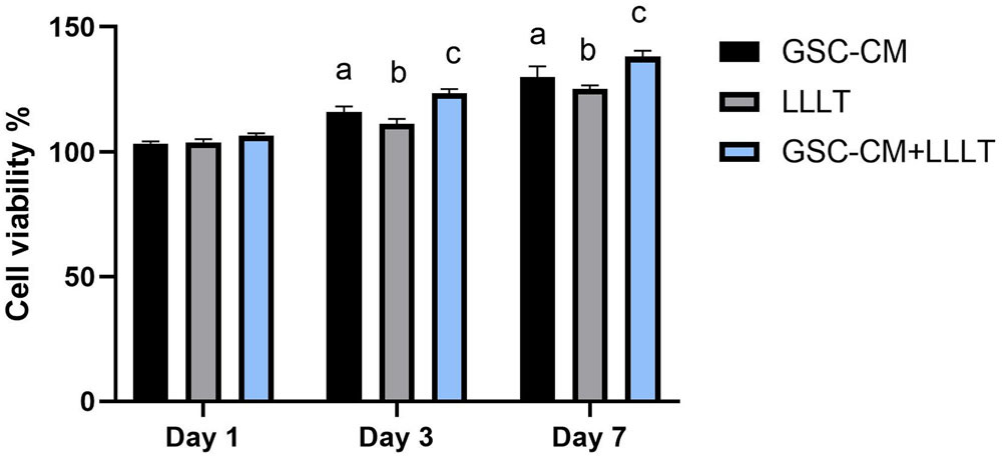
Mean, standard deviation, and comparative statistics of cell viability percentage at Days 1, 3, and 7. Different letters are used to denote significance (*p* < 0.05).

The statistical analysis showed that there were no significant differences (*p* > 0.05) between the treatment groups on Day 1, but significant differences (*p* < 0.05) emerged on Days 3 and 7. On Days 3 and 7, the most pronounced effects were observed in the (GSC‐CM + LLLT) group (*p* < 0.05) compared to the other groups. The mean values of cell viability and multiple comparisons after 1, 3, and 7 days are shown in Figure 2.

### 3.3 Mineralization Assay

**Figure 3:**
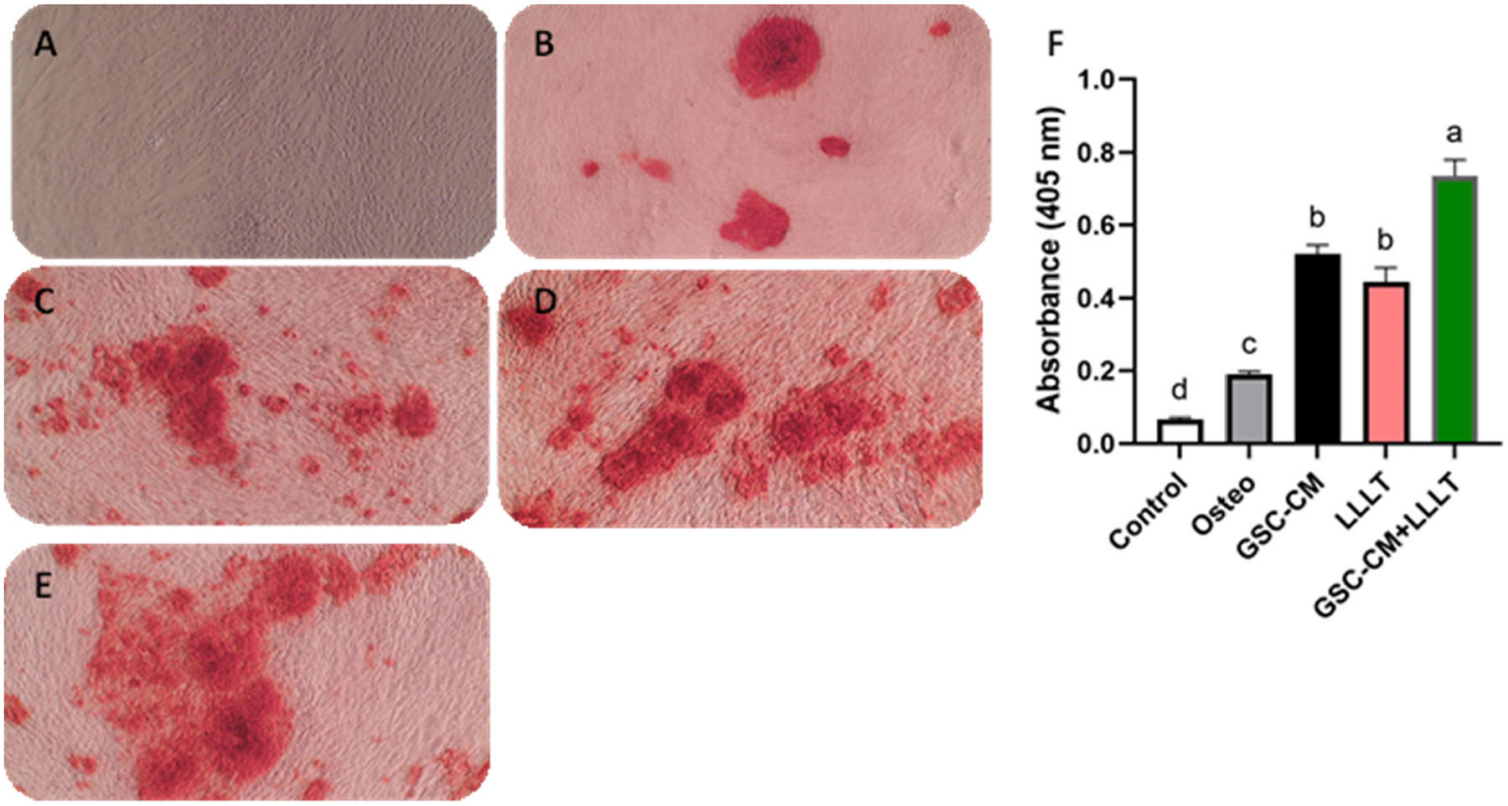
Matrix mineralization as determined by AR staining after 2 weeks of osteogenic induction in the (A) control group, (B) osteogenic group, (C) GMS‐CM group, (D) LLLT group, and (E) GMS‐CM + LLLT group (×100 original magnification). (E) Bar chart of the mean and standard deviation of the AR staining absorbance. Bars labeled with different lowercase letters indicate statistically significant differences (*p* < 0.05) between groups based on post hoc multiple comparisons. Bars sharing the same letter are not significantly different.

AR staining showed that both GSC‐CM and LLLT promoted mineralization to some extent. Nevertheless, their combination seems to have a synergistic effect, resulting in the highest level of mineralization. Quantitative analysis revealed that the control group differed significantly (*p* < 0.05) from the osteogenic, GSC‐CM, LLLT, and (GSC‐CM + LLLT) groups. Additionally, there were significant differences (*p* < 0.05) between the osteogenic group and the other treatment groups (GSC‐CM, LLLT, and GSC‐CM + LLLT). Finally, while there was no significant difference (*p* > 0.05) between GSC‐CM and LLLT alone, both interventions differed significantly (*p* < 0.05) from the combination treatment (GSC‐CM + LLLT), which had the greatest AR absorbance. These findings suggest that the treatments have distinct effects on the outcome measured by the AR assay (Figure 3).

### 3.4 RT‒qPCR

**Figure 4:**
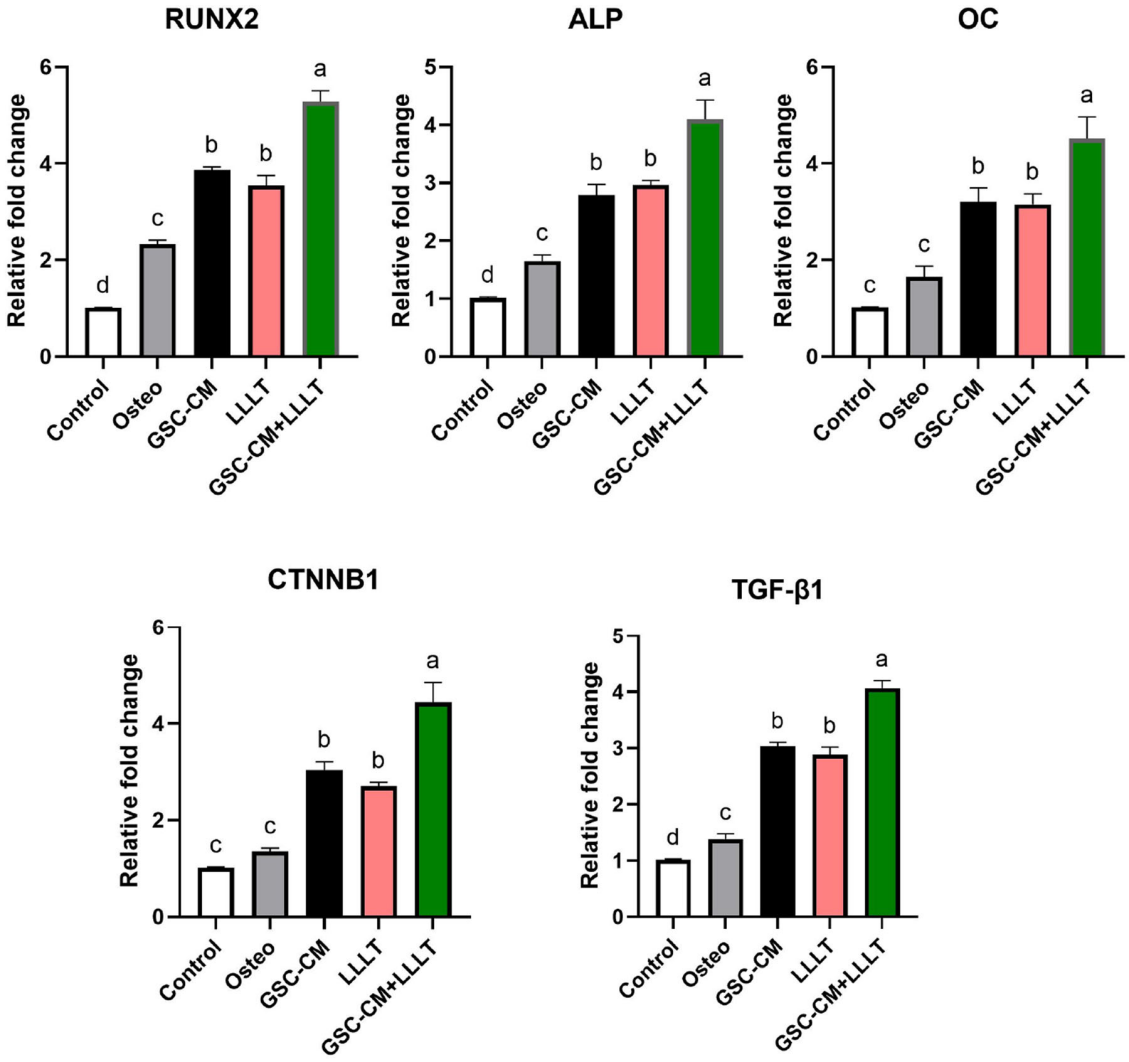
Comparative Analysis of Gene Expression Changes Among Different Treatment Groups. The figure represents the relative fold changes in gene expression for *RUNX2, ALP, OC, CTNNβ1*, and *TGF‐β1* among the various treatment groups: Control, Osteo, GSC‐CM, LLLT, and GSC‐CM + LLLT. Bars indicate mean relative expression levels normalized to the control group, with error bars representing the standard error of the mean. Bars labeled with different lowercase letters indicate statistically significant differences (*p* < 0.05) between groups based on post hoc multiple comparisons. Bars sharing the same letter are not significantly different.

For *RUNX2*, *ALP*, and *OC*, the GSC‐CM + LLLT treatment had the greatest relative fold change, suggesting a synergistic effect of the combined treatments on the expression of these genes associated with bone formation and mineralization. For *CTNNB1*, LLLT treatment alone had the greatest effect on CTNNB1 expression, although the combined GSC‐CM + LLLT treatment also significantly increased CTNNB1 expression compared to that in the control and GSC‐CM alone groups. For *TGF‐β1*, both LLLT alone and combined GSC‐CM + LLLT treatment had similarly high relative fold changes, suggesting that LLLT may be a potent inducer of *TGF‐β1* expression (Figure 4).

## 4 Discussion

The primary objective of periodontal treatment is to restore the compromised structures that support the tooth to its original shape, structure, and functionality (Qiu et al. 2020). This study's objective was to assess the effect of GSC‐CM and LLLT, alone or combined, on the viability and osteogenic performance of PDLSCs. The synergy between GSC‐CM and LLLT underscores the multidisciplinary nature of regenerative medicine, where various approaches and technologies are leveraged to optimize cellular responses and tissue healing by targeting specific signaling pathways.

GSCs were chosen as an accessible source of CM because they are known to secrete an array of growth factors that can have paracrine effects on neighboring cells (Praveen Kumar et al. 2019). This ability is crucial because these secreted factors enhance cell proliferation, migration, and tissue repair. Furthermore, GSCs have shown promise as a source of SCs for bone regeneration. This potential is underscored by research (Wang et al. 2011; Xu et al. 2014) indicating its effectiveness in this area. The contribution of GSCs to bone regeneration is especially remarkable due to the intricate composition of bone tissue and the difficulties associated with its repair and regeneration. The role of GSCs in periodontal regeneration is a topic that requires additional investigation.

Building on these principles, the evaluation of GSCs involves determining their ability to effectively integrate with diverse treatment modalities. Research has demonstrated that the concurrent use of GSC‐CM and low‐level laser therapy (LLLT) has synergistic effects on both the survival and osteogenic differentiation of periodontal ligament stem cells (PDLSCs). The combined application of GSC‐CM and LLLT significantly surpassed the effects of either treatment alone, underscoring the advantage of therapeutic combinations in improving tissue regeneration. In the current study, we utilized a 980 nm wavelength for LLLT based on its well‐documented penetration depth and photobiomodulation effects on hard and soft tissues. Compared to lower wavelengths (e.g., 635–810 nm), 980 nm has demonstrated deeper tissue penetration and a higher affinity for water and is effective for stimulating cellular responses in deeper tissues, including MSCs. Moreover, a comparative study by Hanna et al. (2019) reported that 980 nm was more effective than 808 nm in stimulating Wnt/β‐catenin pathway activation in MSCs, aligning with our mechanistic focus (Hanna et al. 2019). On the other hand, the 100‐fold concentration of the conditioned media was selected based on prior studies demonstrating its efficacy in enhancing bioactive factor delivery to target cells (Qiu et al. 2020).

The study revealed that the viability of PDLSCs, as measured by the MTT assay, did not show any early differences on Day 1. However, the combination of GSC‐CM and LLLT had a distinct and increasingly significant effect on cell viability on Days 3 and 7. It is possible that the combined treatment has cumulative or synergistic effects on PDLSCs, which may improve their viability by preventing cell death and promoting cell growth (Alessio et al. 2017; Saleem et al. 2021). Furthermore, these findings suggest that the combined treatment yields different results than the individual therapies, and this difference becomes more noticeable over time. These results align with those of Hendudari et al. in 2016 (2016), who demonstrated that the combined application of LLLT and human bone marrow SC‐CM significantly enhanced the viability of human dermal fibroblasts compared to the effects of CM and LLLT administered separately (Kouhkheil et al. 2018) revealed that the simultaneous use of bone marrow SC‐CM and LLLT synergistically impacted the healing process of infected skin wounds.

The LLLT employed in this study utilized a wavelength of 980 nm, a range spanning from 600 to 1000 nm, which is widely acknowledged to be highly effective in stimulating cellular responses. It was also used at a low energy level of 1.5 J/cm^2^, a parameter that significantly enhances the impact on MSCs (Alhazmi et al. 2023). This positive effect of LLLT can be attributed to its ability to stimulate the release of adenosine triphosphate (ATP), which functions as the principal cellular energy source. By augmenting ATP synthesis within the mitochondria, cells acquire an increased energy reservoir for various cellular processes, such as repair and regeneration (de Freitas and Hamblin 2016). Similar results were found in previous studies exploring the effect of LLLT on MSCs of dental origin (Wu et al. 2023; Asnaashari et al. 2022).

Moreover, LLLT is documented to transiently increase the intracellular reactive oxygen species (ROS) which functions as secondary messengers in cellular signaling pathways. In MSCs, the slight increase in ROS concentrations caused by LLLT, activate signaling pathways. Notably, pathways such as Wnt/β‐catenin and TGF‐β/SMAD, which are examined in our study, are among the key pathways activated by the increased ROS levels caused by LLLT. This elevated ROS also stimulates cell proliferation and osteogenic differentiation (Khalid et al. 2020).

This study evaluated the osteogenic differentiation capability of PDLSCs stimulated with GSC‐CM and LLLT. Compared with the control and osteogenic (positive control) groups, all the intervention groups had a positive impact on mineralization. The combination of GSC‐CM and LLLT resulted in the most significant enhancement of mineralization. This indicates that the combination of GSC‐CM and LLLT had a synergistic effect, resulting in a more pronounced promotion of mineralization than either treatment alone.

Additionally, the expression levels of specific osteogenic markers, including *RUNX2*, *ALP*, and *OCN*, were monitored via RT‒qPCR analysis. The osteogenic differentiation process encompasses three phases: proliferation, maturation, and mineralization. RUNX2 plays a significant role in the migration of MSCs and their subsequent osteogenic differentiation, and ALP is an early indicator of bone formation (Tariq et al. 2019). At the same time, OCN is a critical protein in bone metabolism regulation and mineralization (Komori 2020). The RT‒PCR results suggested that, individually, GMS‐CM and LLLT had similar effects on the expression of these osteogenic genes. The results of a study by Gholami et al. (2022) showed no significant increase in cell proliferation after LLLT, however they reported a statistically significant upregulation in the expression of osteogenic‐related genes and alkaline phosphatase (ALP) activity. These findings are in line with our results, where we observed enhanced osteogenic marker expression and activation of Wnt and TGF‐β/SMAD signaling pathways following 980‐nm LLLT application (Gholami et al. 2022).

In contrast, when the combined therapy was applied, there was significantly greater expression (*p* < 0.05) of the osteogenic genes than when GMS‐CM or LLLT was applied separately. The synergistic effect seen with the combined therapy implies that these two treatments may work together to enhance their benefits regarding osteogenesis.

Gene expression analysis of beta‐catenin (CTNN*β*1) and TGF *β*1 y was also performed to study the Wnt and TGF‐β signaling pathways associated with osteogenic differentiation. The Wnt signaling pathway is critical for various cellular processes, including tissue development, regeneration, and maintenance of cellular functions (Houschyar et al. 2019). Previous research has established the fundamental role of the Wnt pathway in influencing the biological activities of PDLSCs, facilitating stem cell self‐renewal, and enabling multilineage differentiation (Zhang et al. 2017; Liu et al. 2014) through governing the differentiation of PDLSCs into osteoblasts, cementoblasts, and fibroblasts (Wei et al. 2020).

Another important signaling system, the TGF‐β pathway, has various roles in extracellular matrix synthesis, immunological regulation, and tissue repair (Wang et al. 2021). Collagen and fibronectin, which are crucial for maintaining the structural integrity of periodontal tissues, are synthesized through the TGF pathway, which plays a pivotal role in periodontal regeneration. Furthermore, this pathway promotes the differentiation and specialization of osteoblasts and fibroblasts (Fan et al. 2019; Li et al. 2019).

RT‒qPCR analysis of the GSC‐CM data revealed a noteworthy increase in the expression of the CTNNB1 (beta‐catenin) and TGF‐β1 genes in the osteogenic group compared to those in the untreated group. The increase in expression observed indicates that GSC‐CM possesses components that have the capacity to activate the Wnt/β‐catenin and TGF‐β pathways. Furthermore, the capacity of GSC‐CM to stimulate both pathways indicates its potential as a powerful regenerative treatment for repairing periodontal tissue.

Activating the Wnt/β‐catenin pathway is essential for regulating osteoblast development and the formation of bone tissue throughout the osteogenic process (Vlashi et al. 2023). Similarly, the increased activity of the TGF‐β pathway, as indicated by the increased expression of TGF‐β1 and its associated genes, suggested that GSC‐CM may also activate this process. The TGF‐β pathway is known for its crucial role in tissue regeneration, specifically in the formation and repair of bones. The components found in GSC‐CM likely activate TGF‐β1, initiating a series of interconnected events, such as autoregulation and feedback mechanisms, that ultimately result in the upregulation of target gene expression. LLLT was shown to be associated with comparable upregulation of the beta‐catenin (CTNNB1) and TGFB1 genes (Zhang et al. 2018; Bai et al. 2021). This process is associated with the production of reactive oxygen species (ROS), which are influenced by dosage.

Emerging evidence suggests that Wnt and TGF‐β signaling pathways do not act independently, but rather interact in a coordinated and context‐dependent manner to regulate mesenchymal stem cell behavior and osteogenic differentiation. For example, TGF‐β can modulate components of the canonical Wnt pathway by influencing β‐catenin stability and transcriptional activity (Gumede et al. 2024). Conversely, Wnt signaling has been shown to modulate TGF‐β/SMAD activity, particularly in osteogenic contexts, enhancing the expression of downstream targets involved in matrix production and mineralization (Tahoori et al. 2025).

The combined effect of GSC‐CM and LLLT had a significantly greater effect on gene expression than either agent alone. Both GSC‐CM and LLLT may activate specific cell signaling pathways. Overall, these genes might converge on common pathways or trigger unique cascades that promote cell survival and proliferation. These pathways could involve growth factors, cytokines, or intracellular signaling molecules. GSC‐CM may contain factors activating this pathway, while LLLT can modulate its activity.

While our study did not specifically quantify individual growth factors in the GSC‐CM, previous proteomic analyses of GSC‐CM have identified a range of bioactive molecules that may contribute to the observed effects. Notably, GSC‐CM has been shown to contain TGF‐β1, VEGF, IGF‐1, BMP‐2, and Wnt‐related proteins, which are well‐documented to influence osteogenic differentiation and the activation of Wnt and TGF‐β signaling pathways (Zhang et al. 2012).

The current results suggest that GSC‐CM and LLLT may influence the Wnt/β‐catenin and TGF‐β pathways. However, it is important to note that the present data, including RT‐PCR analysis of CTNNβ1 and TGFβ1, provide preliminary insights and are not sufficient to confirm the activation of the Wnt/β‐catenin pathway. Further studies involving additional markers and protein‐level analyses are required to validate these findings. Moreover, this study focused on cellular and molecular outcomes, but the clinical relevance and translatability of these findings to actual periodontal tissue regeneration in patients remain to be established. Clinical trials are required to assess the safety and efficacy of this combination therapy in humans.

## 5 Conclusions

This study demonstrated that the combination of GSC‐CM and LLLT significantly impacts the viability and osteogenic differentiation potential of PDLSCs. This combined effect was notably more pronounced than that of GSC‐CM or LLLT alone.

## Author Contributions

Conceptualization: Mohammed Y. Aljabri, Yaser A. Alhazmi, and Mohamed Shamel. Formal analysis: Salah A. Elsayyad. Investigation: Salah A. Elsayyad and Mohamed Shamel. Methodology: Salah A. Elsayyad and Mohamed Shamel*.* Supervision: Mohammed Y. Aljabri, Yaser A. Alhazmi, and Shehabeldin M. Saber. Writing – original draft: Mohamed Shamel. Writing – review and editing: Yaser A. Alhazmi, Mohamed Shamel, and Shehabeldin M. Saber. All authors have read and agreed to the published version of the manuscript.

## Ethics Statement

All experiments were carried out in accordance with the guidelines of the World Medical Association Declaration of Helsinki and were approved by the research ethics committee of the Faculty of Dentistry of The British University in Egypt (approval FD BUE REC 23‐036).

## Consent

Informed consent was obtained from all the subjects involved in the study.

## Conflicts of Interest

The authors declare no conflicts of interest.

## Data Availability

The data that support the findings of this study are available from the corresponding author upon reasonable request.
